# ADHD, Methylphenidate, and Childhood Epilepsy

**DOI:** 10.15844/pedneurbriefs-30-6-1

**Published:** 2016-06

**Authors:** Rahul Sharma, Sigita Plioplys

**Affiliations:** 1 Child and Adolescent Psychiatry, Ann & Robert H. Lurie Children’s Hospital of Chicago, Chicago, IL; 2 Departments of Psychiatry and Behavioral Sciences Northwestern University Feinberg School of Medicine, Chicago, IL

**Keywords:** ADHD, Epilepsy, Methylphenidate

## Abstract

Investigators from the Department of Functional Neurology, Epileptology and Epilepsy Institute (IDEE), and the Lyon’s University Hospital examined the clinical determinants of ADHD severity in children with epilepsy (CWE) along with the response to treatment with methylphenidate (MPH).

Investigators from the Department of Functional Neurology, Epileptology and Epilepsy Institute (IDEE), and the Lyon’s University Hospital examined the clinical determinants of ADHD severity in children with epilepsy (CWE) along with the response to treatment with methylphenidate (MPH). A total of 167 patients, aged 6-16, with ADHD and epilepsy entered into a multi-center prospective observational study and were followed over a 12-16-week period. Baseline severity and evolution of ADHD symptoms were assessed using the ADHD Rating Scale-IV. Each subject was assessed for ADHD severity at baseline, response to MPH, and risk of seizures aggravation in those who received MPH. MPH was administered with a dosage of 0.3-1.0mg per kilogram either two to three times per day (short-acting MPH) or once per day (slow-acting MPH). At baseline, 61 out of 167 patients received MPH, whereas the remainder were followed without specific pharmacological intervention. Epilepsy was considered to be well controlled in 91 patients at study entry. Among these patients, 6 demonstrated relapse during follow-up, with 4 being in the MPH treatment group. At study conclusion, 41 subjects treated with MPH and 39 subjects who did not receive pharmacological intervention demonstrated a >25% reduction in ADHD Rating Scale-IV total score. [1]

COMMENTARY. Up to 40% of CWE have comorbid ADHD [2] and both conditions are associated with impairment in quality of life and cognitive functioning [3]. However, factors associated with ADHD development, severity, and response to MPH treatment in this population are not well investigated.

Similar to other studies [3, 4], this study demonstrated no significant association between the occurrence and severity of ADHD symptoms and underlying epilepsy syndrome, severity of epilepsy, or the use of antiepileptic drugs. Despite a relatively large overall sample and prospective study design, this sample included a heterogeneous group of subjects with an uneven distribution of epilepsy types/syndromes and a broad range of antiepileptic drugs in each group. This factor limits meaningful interpretation of the study findings regarding lack of association between the epilepsy related factors and development of ADHD.

This study provides support for efficacy and safety of MPH use in CWE due to statistically significant decrease of ADHD symptoms in 75% of treated subjects. Proportion of seizure relapse was not significantly different between the subjects treated with MPH compared to those who were not. However, neither initiation of MPH or follow up assessment used randomized controlled approach, thus may have been affected by the selection bias amongst other limitations. In addition, there was also a lack of EEG data gathered at baseline or follow-up to assess the subclinical ictal abnormalities and changes during the course of treatment.

To further advance this very important question of the specific association of ADHD and epilepsy as well as efficacy and safety of treatment with MPH, we need to have more rigorous evidence obtained from the randomized double blind controlled treatment studies.
